# Time-course of exercise and its association with 12-month bone changes

**DOI:** 10.1186/1471-2474-10-138

**Published:** 2009-11-12

**Authors:** Riikka Ahola, Raija Korpelainen, Aki Vainionpää, Juhani Leppäluoto, Timo Jämsä

**Affiliations:** 1 Department of Medical Technology, Institute of Biomedicine, University of Oulu, Oulu, Finland; 2 Department of Sport and Exercise Medicine, Deaconess Institute of Oulu, Oulu, Finland; 3 Institute of Health Sciences, Unit of General Practice, University of Oulu, Oulu, Finland; 4 Department of Physical Medicine and Rehabilitation, Seinäjoki Central Hospital, Seinäjoki, Finland; 5 Department of Physiology, Institute of Biomedicine, University of Oulu, Oulu, Finland; 6 Department of Radiology, Oulu University Hospital, Oulu, Finland

## Abstract

**Background:**

Exercise has been shown to have positive effects on bone density and strength. However, knowledge of the time-course of exercise and bone changes is scarce due to lack of methods to quantify and qualify daily physical activity in long-term. The aim was to evaluate the association between exercise intensity at 3, 6 and 12 month intervals and 12-month changes in upper femur areal bone mineral density (aBMD) and mid-femur geometry in healthy premenopausal women.

**Methods:**

Physical activity was continuously assessed with a waist-worn accelerometer in 35 healthy women (35-40 years) participating in progressive high-impact training. To describe exercise intensity, individual average daily numbers of impacts were calculated at five acceleration levels (range 0.3-9.2 *g*) during time intervals of 0-3, 0-6, and 0-12 months. Proximal femur aBMD was measured with dual x-ray absorptiometry and mid-femur geometry was evaluated with quantitative computed tomography at the baseline and after 12 months. Physical activity data were correlated with yearly changes in bone density and geometry, and adjusted for confounding factors and impacts at later months of the trial using multivariate analysis.

**Results:**

Femoral neck aBMD changes were significantly correlated with 6 and 12 months' impact activity at high intensity levels (> 3.9 *g*, *r *being up to 0.42). Trochanteric aBMD changes were associated even with first three months of exercise exceeding 1.1 *g *(*r *= 0.39-0.59, *p *< 0.05). Similarly, mid-femoral cortical bone geometry changes were related to even first three months' activity (*r *= 0.38-0.52, *p *< 0.05). In multivariate analysis, 0-3 months' activity did not correlate with bone change at any site after adjusting for impacts at later months. Instead, 0-6 months' impacts were significant correlates of 12-month changes in femoral neck and trochanter aBMD, mid-femur bone circumference and cortical bone attenuation even after adjustment. No significant correlations were found at the proximal or distal tibia.

**Conclusion:**

The number of high acceleration impacts during 6 months of training was positively associated with 12-month bone changes at the femoral neck, trochanter and mid-femur. These results can be utilized when designing feasible training programs to prevent bone loss in premenopausal women.

**Trial registration:**

Clinical trials.gov NCT00697957

## Background

Exercise during youth and adolescence positively affects peak bone mass, while exercise during adulthood can maintain bone mass and mechanical competence and can potentially prevent women from osteoporosis and fragility fractures [1-3]. In particular, impact exercise that induces high strains at high rates in the bone has been found to promote bone strength [4,5]. In exercise interventions with healthy premenopausal women, a 1-3% net gain in bone mineral density has been observed at mechanically loaded sites in comparison with controls [6-8]. In our previous study, we found that supervised high-impact training resulted in significant bone density gains in the proximal femur and positive changes in bone geometry [9,10]. Furthermore, the intensity of exercise measured from the acceleration signal was associated with bone changes [11,12].

Despite the evidence that suggests that exercise can significantly influence bone properties, knowledge of the time course of exercise and bone changes is scarce. Typically, exercise interventions with premenopausal women last 6-12 months, because bone increments are considered to be slow [6]. The role of the first months of exercise in the bone change is unclear, and it would be essential to know how the intensity of the exercise during the first months of training affects bone changes at 12 months.

Currently, accelerometers as portable, cheap and light-weight are widely used to measure daily physical activity in exercise studies [13]. When studying the relationship of exercise and bone health, exercise intensity and bone loading can be measured from acceleration peak amplitude [14]. Despite the objective measurement of exercise time and intensity, there are challenges related to compliance, data reduction and interpretation in long-term continuous measurements [15]. In our previous study we developed the first device that could measure the daily intensity of impacts at the waist over a long period of time [16]. This accelerometer was used to continuously measure daily physical activity in healthy premenopausal women who were participating in a 12-month population-based exercise trial [9,11]. Using this technology, we previously calculated an average for the 12 months to describe exercise. We now analyzed more deeply the course of the exercise and calculated the average for the first three months and six months as well.

The aim was to evaluate the association between exercise intensity at 3, 6 and 12 month intervals and 12-month changes in upper femur BMD and mid-femur geometry during high-impact exercise in premenopausal women. The general purpose was to obtain new information for adjusting exercise prescriptions.

## Methods

The subjects were 35 healthy women (age 35-40 years, mean 38.3, SD 1.9) who participated in impact exercise training for 12 months [9]. Average height was 162.9 (6.0) cm and BMI was 25.5 (4.6) kg/m^2^. At baseline, they were not participating in impact-type exercises or long-distance running more than three times a week. Mean calcium intake was 1101.1 (532.7) mg per day as assessed from a dietary questionnaire, and 21.2% reported smoking and 39.4% use of oral contraceptives [11].

Daily physical activity was measured both during exercise training and habitual living. The study protocol was approved by the Ethical Committee of the Northern Ostrobothnia Hospital District, Oulu, Finland, and all participants gave written informed consent. The procedure of the study was in accordance with the Declaration of Helsinki.

### Exercise program

The group training (step aerobic) was supervised by a physiotherapist and carried out three times a week for 12 months as previously presented [9,11]. The 60-minute training workout consisted of a warm-up period, high-impact training and a cool-down period. The progressive high-impact period included versatile movements, such as step aerobic patterns, stamping, jumping, and running. The intention of the impact exercise was to create nonhabitual bone strains in order to enhance bone mechanical competence in the lower extremities. The programs were modified bimonthly to become progressively more demanding by including higher jumps and drops. After 3 months of training, one step bench (height 10 cm) was used to enhance the impact effect, and after 6 months, two to three benches were used (height 20-30 cm in total). Compliance to exercise program was 1.4, 1.3 and 1.0 times a week in 0-3, 0-6 and 0-12 months, respectively. Additionally, the participants were given a home program (10 min daily), which consisted of patterns of exercise similar to those in the supervised sessions. Weekly compliance of the home program was on average 2.4 times per week.

### Physical activity measurements

Physical activity was measured with an accelerometer (body movement monitor, Newtest Ltd., Oulu, Finland). The monitor recorded vertical acceleration peaks up to 9.2 *g *(*g*, acceleration of gravity 9.81 m·s^-2^) with a threshold of 0.3 *g*. All subjects were asked to carry the monitor on a belt close to the iliac crest during all waking hours for 12 months. The device has been described in more detail previously [16]. Briefly, the monitor was designed for physical activity measurements over a long period of time. It gathered the data at the sampling rate of 400 Hz, filtered, pre-analyzed and classified according to peak acceleration. Using this data reduction method of classification, the continuous measurement time was extended to several weeks. The reduced data were transferred into a server computer approximately every second week. Compliance was checked from the accelerometer data and individual average daily distribution of impacts was calculated for the analysis for the days, when the monitor was worn.

The average number of daily acceleration peaks (impacts) was first analyzed at the 32 acceleration levels from 0.3 to 9.2 *g *as given by the device, 0 *g *corresponding to standing (acceleration of gravity 1 *g *subtracted). This resulted in a 32-level histogram of impacts according to their peak acceleration value. To simplify accelerometer output, we then combined the numbers of impacts in these 32 levels to five levels using summation to describe exercise intensity: 0.3-1.0 *g *(e.g., walking), 1.1-2.4 *g *(e.g., stepping), 2.5-3.8 *g *(e.g., jogging), 3.9-5.3 *g *(e.g., running and jumping), and 5.4-9.2 *g *(e.g., jumping and drop-jumping). We have used these five levels in our previous study [11]. Finally, we calculated the average daily number of impacts for each individual during 0-3, 0-6, and 0-12 months at these five acceleration levels.

We have tested the precision and accuracy of the accelerometer-based method with a three-dimensional prototype of the body movement monitor [17]. The reproducibility error as the root-mean square coefficient of variation (CV_RMS_) was 4.0%. The peak acceleration values had a high correlation (*r *= 0.989, n = 572 recordings) with the values obtained simultaneously using a standard optical motion analysis system [18]. The acceleration values were also significantly correlated with peak ground reaction force measured with a force plate (Pearson's correlation coefficients *r *= 0.735 for the peak acceleration and *r *= 0.937 for the area under the acceleration peaks; n = 462 recordings), when the acceleration values were multiplied by body weight [11]. The 3D-prototype and the 1D device used in the current study had a high correlation in simultaneous measurements during exercise training (*r *= 0.971, n = 41 subjects) [11].

### Bone measurements

Bone measurements were performed at baseline and after 12 months. Areal bone mineral density (aBMD, g/cm^2^) was measured using dual x-ray absorptiometry (DXA; Hologic Delphi QDR, Bedford, MA, USA) at the left proximal femur. The femoral neck and trochanter were analysed separately. The same operator performed all scanning and analyses. The cross-sectional geometry of the mid-femur, proximal tibia, and distal tibia was assessed bilaterally as previously described with a spiral quantitative computed tomography (QCT) scanner (Siemens Somatom Emotion, Siemens GmbH, Munich, Germany), and the mean of both legs was used in the analysis [10]. Three cross-sectional scans were made, one at mid-femur (50% of the estimated bone length from the distal endplate of the femur), one at proximal tibia (67% from the distal endplate of the tibia) representing cortical bone and one at distal tibia (5%), representing trabecular bone. The scan lines were adjusted using the scout view of the scanner software. Subject positioning was standardized, and one qualified radiographer performed all measurements. The slice thickness was 3 mm and pixel size was 0.34 × 0.34 mm^2^. The images were saved in DICOM format and analysed using the GEANIE 2.1 bone analysis software (Bonalyse Ltd., Jyväskylä, Finland). The image analysis was based on Hounsfield Units (HU), which describe the x-ray attenuation of each voxel of the image, characterizing the local density. Ranges from -250 to 5 HU, from 5 to 220 HU and from 220 to 3000 HU were used to separate fat, muscle and bone from each other, respectively. Cortical bone was separated from trabecular bone using a threshold of 450 HU. Measurements from the mid-femur and proximal tibia included bone periosteal circumference (mm), cortical cross-sectional area (CSA, mm^2^), cortical attenuation (HU), mean cortical thickness (mm), maximum and minimum cortical cross-sectional moment of inertia weighted by HU (CSMI, HU cm^4^), and muscle CSA (mm^2^). From the distal tibia, trabecular attenuation (HU) was measured as a relative measure of trabecular bone mineral density.

The DXA scanner was calibrated daily by bone phantoms (Hologic, Bedford, MA, USA) for quality assurance, and no evidence of machine drift appeared during this study. The CV of the DXA measurement in the laboratory has previously been found to be 0.5% [19]. QCT calibration quality assurance was performed daily according to the manufacturer's recommendations. Reproducibility of the QCT measurements was tested from the duplicate measurement of 15 subjects. CV_RMS _for distal tibia in our laboratory was 1.2%, varied between 0.2% (minimum CSMI) and 0.5% (cortical thickness) for mid-femur, and between 0.5% (circumference) and 1.5% (maximum CSMI) for proximal tibia [20].

### Statistical analysis

The data were analysed using SPSS statistical package (SPSS 16.0 for Windows, SPSS, Chicago, IL, USA). We calculated Pearson's correlation coefficients (*r*) between average daily numbers of impacts in 0-3, 0-6 and 0-12 months and 12-month %-changes in the proximal femur aBMD and bone geometry. Correlations were calculated separately for values of each subject at each level. The Benjamini-Hochberg procedure was used to correct *p*-values for multiple comparisons [21,22]. A corrected *p*-value of < 0.05 was considered statistically significant.

We used multiple linear regression to determine the contribution of number of impacts at different acceleration levels to 12-month bone changes at each measured site. The number of impacts at each acceleration level was entered separately into the models. In addition, separate models were built for 0-3, 0-6 and 0-12 months' periods of exercise. The number of impacts, baseline weight, weight change, baseline bone measurement value, compliance to exercise, and calcium intake were entered into the model using stepwise procedure. Then, a two-level hierarchical model was built to adjust also for impacts at later months of the trial using 0-3 or 0-6 months' impacts as explanatory variables, the number of impacts at 3-12 months or 6-12 months being entered into the model in the second hierarchical block.

## Results

**Figure 1:**
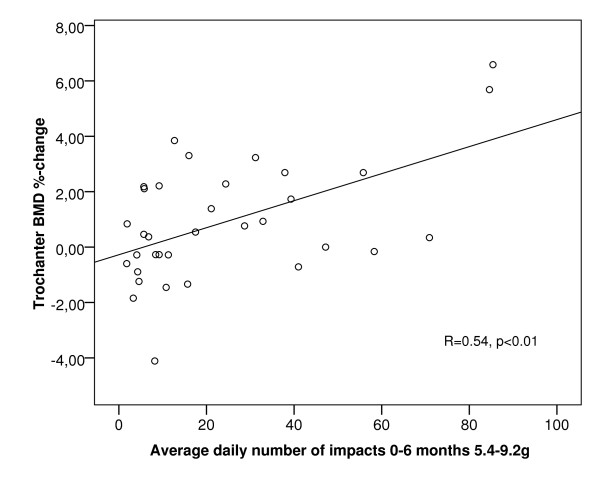
**Correlation between daily number of high impacts at 0-6 months and 12-month trochanter aBMD change**. A scatter plot between 0-6 month average daily numbers of impacts at 5.4-9.2 *g *and trochanteric BMD %-change. N = 34 (DXA data was missing from one subject). *r *= 0.54, *p *< 0.01.

**Figure 2:**
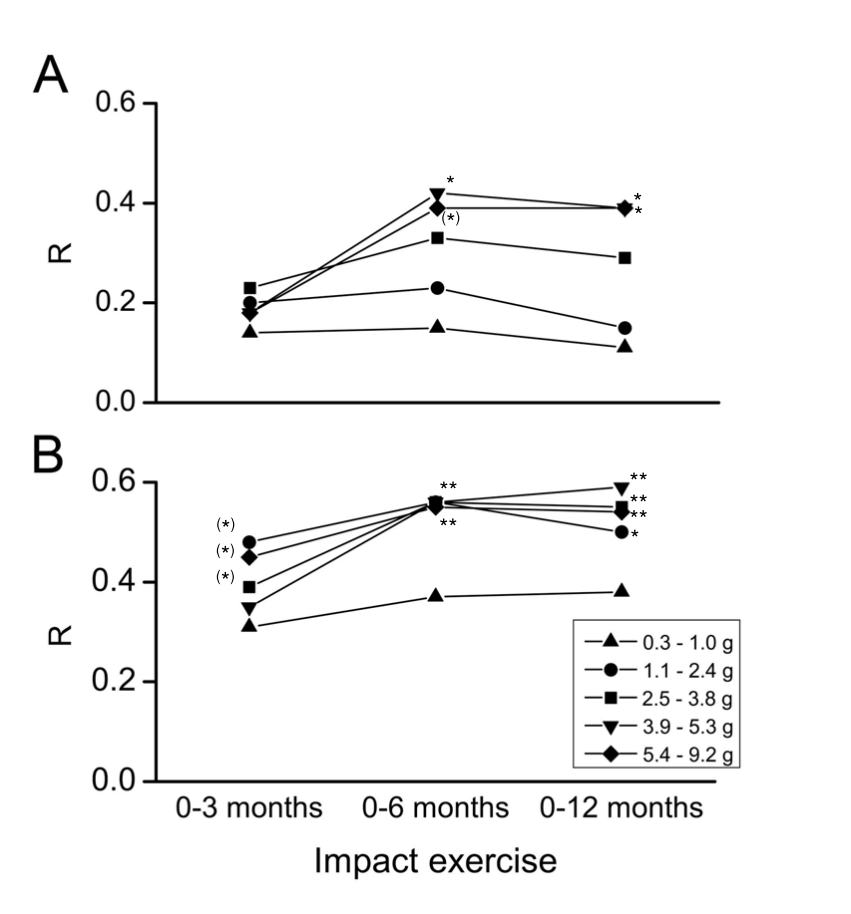
**Correlation between impact exercise at different time intervals and 12-month aBMD changes in proximal femur**. Pearson's correlation coefficients (*r*) between the impact exercise at different acceleration levels in 0-3, 0-6 and 0-12 months, and yearly aBMD changes A) in the femoral neck, and B) trochanter. N = 34 (DXA data was missing from one subject), *g *= acceleration of gravity (9.81 m·s^-2^), * *p *< 0.05, ** *p *< 0.01 after Benjamini-Hochberg corrections, ^(^*^) ^the association did not remain significant after adjustment for impacts at later months in multivariate analysis.

The average daily numbers of impacts at different acceleration levels during 0-3, 0-6 and 0-12 months are presented in Table 1. A scatter plot between 12-month trochanteric aBMD change and daily number of impacts at > 5.4 g in 0-6 months is shown in Figure 1. Correlation coefficients *r *between the average impact activity data during 0-3, 0-6 and 0-12 months and aBMD changes at the femoral neck and trochanter are presented in Figure 2.

**Table 1:** Average daily numbers of impacts during 0-3, 0-6 and 0-12 months

	0-3 months		0-6 months		0-12 months	
0.3-1.0 *g*	7964.7	(6804.8-9124.5)	7828.2	(6796.4-8860.1)	8833.3	(7809.6-9857.0)
1.1-2.4 *g*	601.5	(477.3-725.7)	615.9	(490.1-741.6)	654.0	(514.6-793.4)
2.5-3.8 *g*	117.3	(88.5-146.2)	147.7	(100.8-194.6)	143.9	(94.0-193.8)
3.9-5.3 *g*	43.6	(32.5-54.7)	55.4	(39.7-71.1)	52.5	(37.7-67.3)
5.4-9.2 *g*	14.3	(9.5-19.2)	23.7	(15.5-31.8)	24.6	(16.5-32.7)

Values are mean (95% confidence interval), *g *= acceleration of gravity (9.81 m·s^-2^)

Average daily numbers of impacts at different acceleration levels during 0-3, 0-6 and 0-12 months of the exercise.

Femoral neck aBMD change was not significantly correlated with impact activity within the first three months, while six months of impact exercise at high intensity levels (> 3.9 *g*) were positively associated with bone change (*r *= 0.42, *p *< 0.05) (Figure 2A). In multivariate analysis after adjusting for impacts at later months and other factors significantly associated with response variables in univariate analyses, the number of 0-6 months' impacts > 3.9 g was still significantly associated with 12-month femoral neck BMD change. The association remained the same with cumulative exercise over a period of 12 months (*r *= 0.39, *p *< 0.05, Figure 2A).

A positive association was found between trochanter aBMD change and exercise during the first three months (*r *= 0.39-0.48, *p *< 0.05) (Figure 2B) in univariate analysis. The significant association disappeared in multivariate analysis after adjusting for impacts at later months. The number of impacts in 0-6 months exceeding 1.1 *g *were correlated with trochanter BMD change (*r *= 0.55, *p *< 0.01, Figure 2B) and this was also confirmed in multivariate analysis. A similar association was found with cumulative exercise over a period of 12 months, and exercise > 1.1 *g *was significantly associated with trochanter BMD change, explaining 25-35% of the change depending on the acceleration level.

Associations between impact activity during 0-3, 0-6 and 0-12 months and 12-month changes in mid-femur cortical bone geometry are presented in Table 2. Changes in mid-femoral cortical geometry were positively associated with impact exercise within the first three months of exercise alone, but this association did not remain in multivariate analyses after adjusting for impacts at 3-12 months. Positive correlations were also found between impacts at 0-6 months and cortical geometry changes (Table 2). In multivariate analysis after adjusting for covariates and impacts at later months, mid-femur circumference and cortical attenuation changes were significantly explained by impact activity. The average daily numbers of impacts from baseline to 12 months significantly explained the changes in mid-femur cortical bone geometry, the acceleration thresholds being 1.1 *g *for cortical thickness, cortical attenuation, and maximal cortical CSMI; 2.5 *g *for bone circumference and 5.4 *g *for cortical CSA. No significant correlations were found at the proximal or distal tibia (data not shown).

**Table 2:** Correlation between the impact exercise at 0-3, 0-6 and 0-12 months and bone geometry changes

			Average daily numbers of impacts at different acceleration levels												
			
	0-3 months					0-6 months					0-12 months				
	
	0.3- 1.0 *g*	1.1- 2.4 *g*	2.5- 3.8 *g*	3.9- 5.3 *g*	5.4- 9.2 *g*	0.3- 1.0 g	1.1- 2.4 *g*	2.5- 3.8 *g*	3.9- 5.3 *g*	5.4- 9.2 *g*	0.3- 1.0 *g*	1.1- 2.4 *g*	2.5- 3.8 *g*	3.9- 5.3 *g*	5.4- 9.2 *g*
*Mid-femur*															
Bone circumference	-0.02	0.17	0.07	0.22	0.45^(^*^)^	0.01	0.21	0.35	0.37	0.45*	0.04	0.23	0.39*	0.41*	0.45*
Cortical CSA	0.08	0.37	0.35	0.35	0.28	0.06	0.33	0.26	0.41^(^*^)^	0.39^(^*^)^	0.12	0.36	0.26	0.41^(^*^)^	0.43*
Cortical attenuation	0.08	0.43^(^*^)^	0.41^(^*^)^	0.52^(^*^)^	0.49^(^*^)^	0.10	0.47^(^*^)^	0.51*	0.54*	0.51*	0.18	0.52*	0.52*	0.54*	0.54*
Max cortical CSMI	0.27	0.38^(^*^)^	0.29	0.34	0.39^(^*^)^	0.29	0.41^(^*^)^	0.39^(^*^)^	0.38^(^*^)^	0.42^(^*^)^	0.38^(^*^)^	0.46*	0.42*	0.43*	0.45*
Cortical thickness	0.04	0.40^(^*^)^	0.33	0.31	0.26	0.00	0.38^(^*^)^	0.30	0.44^(^*^)^	0.39^(^*^)^	0.05	0.41*	0.30	0.44*	0.41*

*g *acceleration of gravity (9.81 m·s^-2^), CSA cross-sectional area, CSMI cross-sectional moment of inertia.

* *p *< 0.05, after Benjamini-Hochberg correction for multiple comparisons

^(^*^) ^the association did not remain significant in multivariate analysis after adjustment for impacts at later months and other confounding factors.

Pearson's correlation coefficients (*r*) between the impact exercise at different acceleration levels in 0-3, 0-6 and 0-12 months, and changes in cortical bone geometry measured with quantitative computed tomography. N = 35

## Discussion

For the first time, using continuous impact exercise monitoring, we found that average daily number of high impacts during six months of training was significantly associated with 12-month aBMD changes at the femoral neck and trochanter area. A positive relationship was also found in bone circumference and cortical bone attenuation in mid-femur.

The results of this study complement our previous studies, in which we calculated correlations using a yearly average of acceleration values with a larger sample size, including also non-exercises [10,11]. These results are in good agreement with previous studies in which five to six months of high-impact exercise were needed to increase aBMD in the femoral neck and trochanter [23-25]. However, in a recent study nine weeks of strength training with plyometric jumping elicited a small, but significant increase in femoral neck BMC, but not in BMD [26]. Our data are also in good accordance with studies in which the time course of bone changes was studied using intermediate aBMD measurements. Bassey and Ramsdale (1994) prolonged their original six-month study for another six months and found that the major changes were obtained during the first six months [23]. Similar results were found in an 18-month resistance training intervention, in which the slope for changes in aBMD from baseline to five months was significantly greater than the slope from 5 to 18 months [27]. However, a linear increase in aBMD at the trochanter, femoral neck, and whole body over 0-6 and 6-12 months was reported in a 12-month jumping plus lower body resistance training study [28]. Similarly, an almost linear increase in femoral neck aBMD was reported when the impact exercise of the original 18-month intervention was continued without supervision for 8 months [4,29]. More recently, in a 12-month site-specific upper and lower body resistance plus jumping program, aBMD increased progressively over time [30].

Previous findings suggest that bone response to exercise is slower at the femoral neck than at the trochanter region [24,28,30,31]. Here, a similar trend was found. The relatively fast response of the trochanter to exercise might be clinically important, because trochanteric fractures are associated with considerably higher morbidity and mortality than femoral neck fractures [28,32,33]. The different responses of the trochanter and femoral neck can be explained by their different loading characteristics [6,19,23]. The trochanter is mainly subjected to tensile forces produced by the hip and gluteus muscles attached to it. Magnitudes and rates of these forces are especially high during the take-off, and the landing phases of jumping may also create high bone strain and strain rate levels. In contrast, the femoral neck is subjected to compressive forces due to weight-bearing, especially during landing. A woman at this age has an average annual reduction of around 0.5% in BMD [34,35]. Considering this, there was only one subject of active exercisers who did not benefit (Figure 1). This supports the effectiveness of impact exercise on aBMD.

Bone strength is influenced not only by its density but also its geometry [36]. Bone adapts to loading by changing its structure to be mechanically appropriate according to its loading environment [37-39]. Exercise has been shown to increase bone cross-sectional dimensions (total circumference, cortical thickness) [10,40] which is achieved by reduced endocortical resorption and (or) greater periosteal apposition [41-43]. This increases cross-sectional moment of inertia and provides greater resistance to bending. A distinct advantage of quantitative computed tomography is its ability to measure cross-sectional geometry and discriminate between trabecular and cortical bone. In this study we showed that six months of exercise were associated with mid-femur cortical bone geometric adaptation. Number of high impacts was even more strongly associated with cortical density evaluated as Hounsfield Units. This is contrary to previous studies, in which the exercise-induced improvement in bone strength has been caused by redistribution of bone rather than remineralisation [40,44]. The relationship found in this study suggests that the higher number of high impacts may help prevent the loss in volumetric BMD.

In contrast to mid-femur, no significant associations were found between the loading characteristics and bone changes at the tibia. It has been proposed that the strain stimulus threshold for bone adaptation varies within individual and between different bones depending on the local strain environment [45-47]. In rat ulna, the strain threshold was found to be largest distally, where strains encountered in daily activities were typically higher and smaller more proximally [45]. Additionally, unusual strain distributions (strain gradients) in the bone may be more osteogenic than high strain magnitudes solely [48,49]. Based on these previous results, the missing loading response at the tibia may be due to the larger strain stimulus threshold at that site. The tibia may have been more accustomed to higher daily loading than was the femur. Thus, it is possible that higher loads would have been needed in the exercise program of the original study to elicit changes in tibia geometry [10]. Additionally, there might be differences in the sensitivity of QCT method to detect changes in bone at different measurement sites.

The sensitivity of DXA to detect changes in bone is limited because of its planar nature, which makes aBMD dependent on bone size, apparent density and projection [50] as well as soft tissue inaccuracies [51]. These inherent properties of DXA measurement may modulate the association observed between bone loading and changes in BMD, and also explain at least to some extent the slight differences observed between the accelerations and QCT and DXA findings.

Because bone cells adapt to habitual loading, one important feature of an exercise program is progression [5]. A progressive exercise program sustains overload and the bone adaptation process. Loads should be increased with time to produce a sufficient stimulus. An exercise program that maintains the same loading for many years would stimulate bone formation only during the first months of training [52]. Our exercise program was progressive since it was modified bimonthly to become more demanding. After the first three months of getting used to training, one step bench was used to enhance impact, and after six months, two or three step benches were used. The progression can be seen in the average daily numbers of impacts as higher numbers at high acceleration levels. However, the increment was not statistically significant mainly because we report cumulative exercise values (0-3, 0-6 and 0-12 months) which overlap.

The time-dependency of bone response to exercise might partly be explained by the interaction between vitamin D and exercise. In our recent paper with the present study sample, increases in vitamin D were found in 6 months followed by decreases in 12 months [53]. However, there was no significant difference in vitamin D level between the high-impact exercise group and a control group over the trial. It was also confirmed that the dietary calcium intake was within recommended levels [53].

There were some limitations in this study. We did not incorporate intermediate bone measurements. Thus, we were not able to analyse the time-course of bone changes. This issue has to be considered in future studies. Instead of true volumetric BMD we used x-ray attenuation that is comparable to volumetric BMD, because we were not able to apply bone density calibration phantoms during the QCT measurements. The impacts were recorded on a daily basis, and we were unable to distinguish single exercises or rest periods. Our device measured only vertical accelerations, and we were not able to quantify other loading directions, which have also been shown to be important [38].

## Conclusion

In this study, we used continuous impact exercise monitoring to determine the association of the time-course of high-impact exercise with bone outcome at 12 months. We conclude that the number of high acceleration impacts during 6 months of training was positively associated with 12-month bone changes at the femoral neck, trochanteric region and mid-femur. The results provide new information for designing optimal and feasible training programs that can prevent bone loss in premenopausal women.

## Competing interests

The authors have a patent application with Newtest Ltd. TJ is also a minor shareholder of Newtest Ltd.

## Authors' contributions

RA contributed to the original idea of the manuscript, data analysis and interpretation of results, drafting the manuscript and manuscript revision. RK contributed to the original idea of the manuscript, design of the study and exercise program, interpretation of results and manuscript revision. AV participated in the design of the study, bone and acceleration measurements, collecting the data, and critically revised the manuscript. JL contributed to the design of the study, was the principal investigator of the original trial, and critically revised the manuscript. TJ had the original idea of the manuscript, and contributed to the design of the study, interpretation of results and critically revised the manuscript. All authors read and approved the final manuscript.

## Pre-publication history

The pre-publication history for this paper can be accessed here:

http://www.biomedcentral.com/1471-2474/10/138/prepub
